# Delayed phase computed tomography angiography ASPECTS predicts clinical outcome and final infarct volume

**DOI:** 10.1371/journal.pone.0239510

**Published:** 2020-09-22

**Authors:** Byung Hoon Lee, Yoon Joon Hwang, Jin Woo Kim

**Affiliations:** 1 Department of Radiology, Inje University Ilsan Paik Hospital, Goyang, Korea; 2 Department of Radiology, Gangnam Severance Hospital, Seoul, Korea; Hospital Dr. Rafael A. Calderón Guardia, CCSS, COSTA RICA

## Abstract

**Background and purpose:**

Non-contrast computed tomography (NCCT) Alberta Stroke Program Early CT Score (ASPECTS) and assessment of collateral flow with multiphase computed tomography angiography (CTA) have been investigated as predictors of clinical outcome in patients with acute ischemic stroke. This study assessed the value of multiphase CTA ASPECTS in predicting final infarction core and clinical outcome in patients undergoing endovascular treatment of acute ischemic stroke.

**Methods:**

We retrospectively studied consecutive patients who underwent multiphase CTA prior to endovascular treatment of acute stroke due to anterior circulation large artery occlusion. Multiphase CTA and final diffusion-weighted imaging (DWI) scans were evaluated by two independent observers for NCCT ASPECTS, acute phase CTA (CTA-AP) ASPECTS, delayed phase CTA (CTA-DP) ASPECTS, and final DWI ASPECTS. Modified Rankin Scale score ≤2 at 3 months was considered a favorable outcome.

**Results:**

A total of 74 patients were analyzed. We found that CTA-DP ASPECTS (*r* = 0.82; 95% CI, 0.73–0.91; p < 0.001) correlated with final DWI ASPECTS better than NCCT ASPECTS (*r* = 0.49; 95% CI, 0.39–0.59) and CTA-AP ASPECTS (*r* = 0.71; 95% CI, 0.64–0.78). Interobserver agreement was higher for CTA-DP ASPECTS (*k* = 0.84). Good CTA-DP ASPECTS was an independent predictor of favorable outcome (odds ratio, 8.71; 95% CI, 3.71–17.3; p < 0.001).

**Conclusion:**

CTA-DP ASPECTS is a reliable predictor of final infarction core and neurological outcome.

## Introduction

In the treatment of acute ischemic stroke due to anterior circulation large vessel occlusion, endovascular treatment has become the standard therapy [1–3]. Effective methods to select appropriate candidates who would benefit from endovascular treatment while avoiding futile attempts at reperfusion are being introduced [4–6]. The Alberta Stroke Program Early CT Score (ASPECTS) on non-contrast CT (NCCT) is widely used for the assessment of early ischemic change [7, 8]. ASPECTS is a semiquantitative scale established to assess the extent of early ischemic changes in patients with acute ischemic stroke of the middle cerebral artery [7]. The territory of the middle cerebral artery is divided into 10 regions, ASPECTS subtracts the regions of early ischemic sign, such as hypoattenuation from the initial score of 10 [7, 8]. However, this has been debated as it is difficult to accurately determine early ischemic change on NCCT and interrater reliability is modest [9]. Recent randomized controlled trials have used the NCCT ASPECTS only as an exclusion criterion [1, 10, 11]. CTA quickly identifies large vessel occlusion and shows early ischemic change better than NCCT [12–14]. In addition, a few studies have shown that CTA ASPECTS can predict final infarct size and clinical outcome better than NCCT ASPECTS [15, 16].

The status of collateral flow is strongly and independently associated with clinical outcome in acute ischemic stroke [17–19]. Multiphase CTA is comprised of non-contrast (NCCT), arterial (CTA-AP), and delayed (CTA-DP) phases and is useful to evaluate collateral status and select patients for endovascular treatment [1, 18]. We hypothesized that multiphase CTA ASPECTS reflects collateral status and thus final infarction volume and also clinical outcome. Therefore, this study aimed to assess the value of each phase in predicting final infarction core and clinical outcome in acute ischemic stroke patients undergoing endovascular treatment.

## Methods

We reviewed the records of consecutive patients with acute ischemic stroke due to anterior circulation large artery occlusion who underwent endovascular treatment between June 2014 and March 2016. The indication for endovascular treatment was an initial National Institute of Health Stroke Scale (NIHSS) score ≥4 within 6 h of symptom onset and confirmation of large artery occlusion on multiphase CTA. Anterior circulation large arteries were defined as the intracranial internal carotid artery (ICA), M1 segment of the middle cerebral artery (MCA), and proximal M2 segment of the MCA. Intravenous tissue plasminogen activator (tPA) was administered (0.9 mg/kg) if indicated. Endovascular thrombectomy was subsequently performed without waiting for the tPA effect. All endovascular treatment was performed under local anesthesia; conscious sedation was administered as necessary. The method of endovascular treatment (stent-retriever thrombectomy, aspiration thrombectomy, or both combined) depended on the treatment situation and preference of the primary operator.

Magnetic resonance imaging (MRI), such as diffusion-weighted imaging (DWI) sequences and time-of-flight angiography, was performed within 36 h in almost all patients who underwent endovascular treatment. Demographic characteristics and NIHSS score were assessed in all patients. The NIHSS score was reappraised within 24 h after treatment and at discharge. Functional outcomes were assessed using the modified Rankin Scale (mRS) at 3 months.

This retrospective study was approved by Ilsan Paik hospital Institutional Review Board. The Clinical Research Ethics Committee waived the need for written informed consent from the participants because the data released from the hospital database were analyzed anonymously.

### Image acquisition

All patients underwent standard NCCT with 5 mm section thickness followed by multiphase CTA with a 64-detector row scanner (Aquilion 64; Toshiba Medical System, Tokyo, Japan). Multiphase CTA was performed in three phases after contrast injection. The first phase (arterial) was obtained from the aortic arch to the skull vertex during the peak arterial phase and was triggered by bolus monitoring. Contrast media (80–100 mL) was injected at a rate of 5 mL/s followed by a 50 mL normal saline chase at a rate of 6 mL/s. Images were acquired with a 0.625 mm section thickness and reconstructed with 3 mm thickness at 1 mm intervals. The first phase was obtained in less than 7 s, and the second and third phases were obtained after respective delays of 4 s each that allowed for table repositioning to the skull base [18]. The third phase CTA was used to determine CTA-DP ASPECTS. MRI (Avanto 1.5T; Siemens, Erlangen, Germany), was performed within 36 h after endovascular treatment; DWI sequences were used to determine the final ASPECTS.

### Outcome measurement

Two board-certified neuroradiologists (J.W.K and B.H.L) independently assessed pretreatment multiphase CTA and final DWI for determination of ASPECTS and assessment of recanalization success on cerebral angiography during endovascular treatment. Disagreement between the two was resolved by consensus. Good ASPECTS was defined as a score from 8 to 10, as in a previously published study [20]. Recanalization success was defined as modified Thrombolysis In Cerebral Ischemia (mTICI) grade 2b or 3 on the final angiogram. Total procedure time was defined as the time interval from femoral artery puncture to acquisition of final angiography. Symptomatic intracranial hemorrhage (sICH) was defined as the presence of a large parenchymal hematoma and NIHSS score increase ≥4 compared to admission. Good outcome was defined as a mRS score ≤2 at 3 months.

### Statistical analysis

Statistical analyses were performed using SPSS software version 20.0 (IBM Corp., Armonk, NY, USA). All categorical variables are presented as numbers with frequency (%); continuous variables are presented as means ± standard deviation or medians with interquartile range. Correlations between pretreatment NCCT ASPECTS, multiphase CTA ASPECTS (CTA-AP and CTA-DP) and final DWI ASPECTS scores were determined using the Spearman correlation coefficient with confidence limits calculated using the Fisher z transformation.

Interobserver agreement for pretreatment CTA and final DWI ASPECTS were estimated using Cohen’s kappa coefficient. The results were interpreted according to the standards for strength of agreement proposed by Landis and Koch as follows: poor agreement, ≤0; slight agreement, 0.1–0.20; fair agreement, 0.21–040; moderate agreement, 0.41–0.60; substantial agreement, 0.61–0.80; and almost perfect agreement, 0.81–1.0 [21]. Univariate associations with good clinical outcome were determined by the Fisher exact test, t-test, or Wilcoxon rank-sum test as appropriate. A multivariate binary logistic regression model was constructed to determine independent predictors of good clinical outcome; all variables that showed potential association in the univariate analyses (p < 0.20) were included. P < 0.05 was considered significant.

## Results

A total of 74 patients who underwent pretreatment multiphase CTA, endovascular treatment of anterior circulation large artery occlusion, and follow-up MRI were included for analysis. Mean patient age was 67.3 ± 14.2 years. Thirty-seven patients were male. Before treatment, the median NCCT ASPECTS was 8 (IQR, 6–10), median CTA-AP ASPECTS was 5 (IQR, 1–8), and median CTA-DP ASPECTS was 6 (IQR, 3–8). Median initial NIHSS score was 15 (IQR, 6–23). Successful recanalization (mTICI 2b–3) was achieved in 65 patients (88%) and mean time from onset to recanalization was 301 ± 172 min. The final median DWI ASPECTS was 6 (IQR, 2–9). Patient clinical characteristics are shown in Table 1.

**Table 1:** Patient demographics and characteristics.

Variable	n = 74
Mean age, years (SD)	67.3 (14.2)
Male, n (%)	37 (50.0)
Hypertension, n (%)	53 (71.6)
Diabetes mellitus, n (%)	19 (25.7)
Atrial fibrillation, n (%)	38 (51.4)
Smoking, n (%)	18 (24.3)
Use of intravenous tPA, n (%)	34 (45.9)
Median initial NIHSS score (IQR)	15 (6–23)
Mean time from onset to recanalization, min (SD)	301 (172)
Recanalization success (mTICI 2b–3), n (%)	65 (88%)
Pretreatment non-contrast CT ASPECTS, median (IQR)	8 (6–10)
Pretreatment CTA-AP ASPECTS, median (IQR)	5 (1–8)
Pretreatment CTA-DP ASPECTS, median (IQR)	6 (3–8)
Follow-up DWI ASPECTS, median (IQR)	6 (2–9)

tPA, tissue plasminogen activator; NIHSS, National Institutes of Health Stroke Scale; IQR, interquartile range; mTICI, modified Thrombolysis In Cerebral Ischemia; ASPECTS, Alberta Stroke Program Early Computed Tomography Score; AP, acute phase; DP, delayed phase; DWI, diffusion-weighted imaging

**Fig 1:**
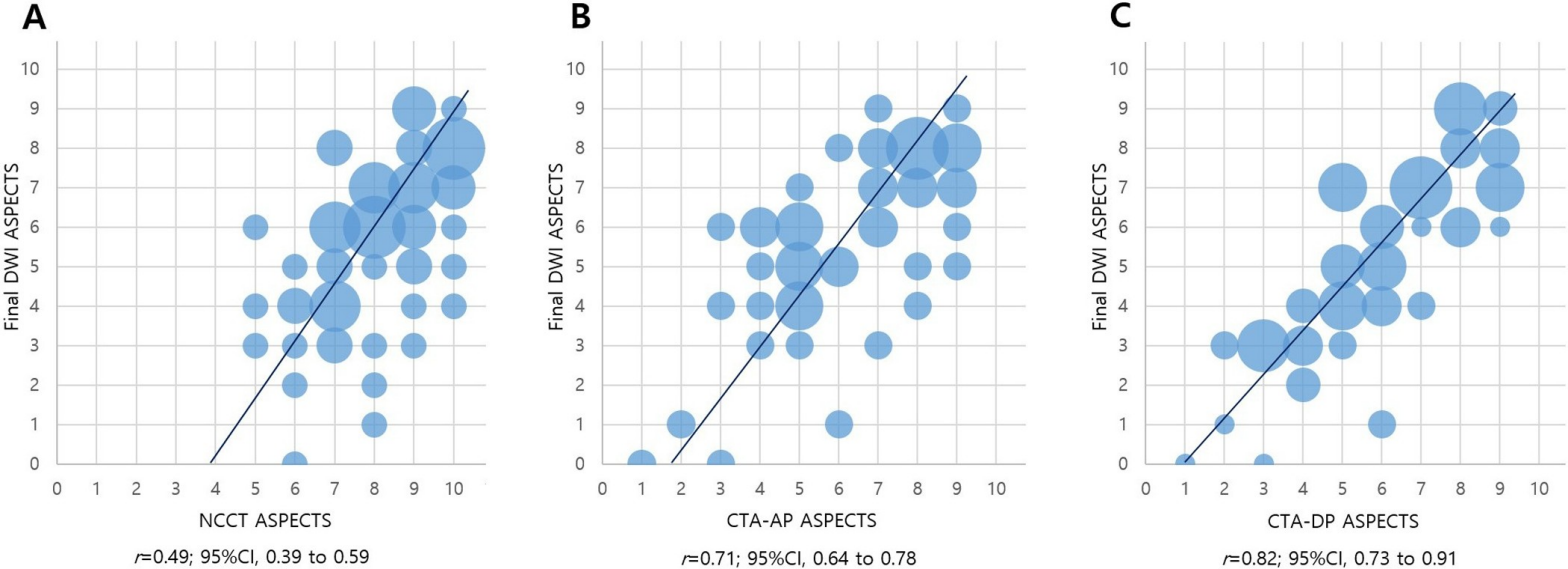
Correlation of ASPECTS on NCCT (A), CTA-AP (B), and CTA-DP (C) with final DWI ASPECTS.

The correlation between CTA-DP ASPECTS (*r* = 0.82; 95% confidence interval (CI), 0.73–0.91; p < 0.001) and final DWI ASPECTS was greater than that of NCCT ASPECTS (*r* = 0.49; 95% CI, 0.39–0.59) or CTA-AP ASPECTS (*r* = 0.71; 95% CI, 0.64–0.78) (Fig 1). Furthermore, the cases of disagreement between the two observers in NCCT ASPECTS, CTA-AP ASPECTS, and CTA-DP ASPECTS were 21, 9, and 7 cases, respectively. The interobserver agreement was classified as almost perfect for CTA-DP ASPECTS (*k* = 0.84) and CTA-AP ASPECTS (*k* = 0.81). Interobserver agreement for NCCT ASPECTS was classified as moderate (*k* = 0.58).

Factors predicting favorable outcome (mRS 0–2 at 3 months) in univariate analysis were younger age, nonhypertension, nonsmoking, low initial NIHSS score, short time from stroke onset to femoral puncture, more successful recanalization, low NIHSS score at 24 h, and good NCCT, CTA-AP, and CTA-DP ASPECTS. In multivariate analysis, good CTA-DP and CTA-AP ASPECTS (odds ratio, 4.21; 95% CI, 2.33–9.01; p = 0.021), age (odds ratio, 0.98; 95% CI, 0.93–0.99; p < 0.001), initial NIHSS score (odds ratio, 0.95; 95% CI, 0.89–0.99; p < 0.001), NIHSS score at 24 h (odds ratio, 0.91; 95% CI, 0.86–0.97; p = 0.015), and successful recanalization (odds ratio, 3.52; 95% CI, 1.11–7.03; p = 0.027) remained independent predictors of favorable clinical outcome. Good CTA-DP ASPECTS (odds ratio, 8.71; 95% CI, 3.71–17.3; p < 0.001) was the best predictor of favorable clinical outcome (Table 2).

**Table 2:** Univariable and multivariable analyses for favorable outcome.

	Univariable analysis			Multivariable analysis	
	mRS 0–2 (n = 39)	mRS 3–6 (n = 35)	*p* value	Odds ratio (95% CI)	*p* value
Age, years	64.8 (11.3)	71.8 (10.7)	<0.001	0.98 (0.93–0.99)	<0.001
Male	51%	49%	0.849	-	-
Hypertension	64%	80%	0.004	0.99 (0.71–1.53)	0.405
Diabetes mellitus	26%	26%	0.947	-	-
Atrial fibrillation	56%	46%	0.132	0.87 (0.81–1.24)	0.955
Smoking	18%	31%	0.013	1.10 (0.69–2.04)	0.580
Use of intravenous tPA	49%	43%	0.233	-	-
Initial NIHSS score	13 (6–20)	17 (10–23)	<0.001	0.95 (0.89–0.99)	<0.001
Time from onset to recanalization, min	290 (±152)	315 (±161)	0.028	0.99 (0.91–1.05)	0.336
Recanalization success	92%	83%	0.004	3.52 (1.11–7.03)	0.027
sICH	10%	14%	0.56	-	-
NIHSS at 24 h	5 (0–10)	13 (5–23)	<0.001	0.91 (0.86–0.97)	0.015
Good NCCT ASPECTS	75%	63%	0.010	1.31 (0.68–2.27)	0.375
Good CTA-AP ASPECTS	37%	28%	<0.001	4.21 (2.33–9.01)	0.021
Good CTA-DP ASPECTS	59%	25%	<0.001	8.71 (3.71–17.3)	<0.001

mRS, modified Rankin Scale; CI, confidence interval; tPA, tissue plasminogen activator; NIHSS, National Institutes of Health Stroke Scale; sICH, symptomatic intracranial hemorrhage; ASPECTS, Alberta Stroke Program Early Computed Tomography Score; AP, acute phase; DP, delayed phase

## Discussion

Our results demonstrate that CTA-DP ASPECTS is both a reliable tool to predict final infarct score and an independent predictor of clinical outcome. NCCT is commonly used to detect early ischemic parenchymal change and exclude acute hemorrhage in the management and evaluation of acute ischemic stroke patients [7, 8]. Although the NCCT ASPECTS is often used to quantitatively evaluate ischemic lesions, NCCT has difficulty detecting early ischemic change and tends to underestimate infarct score [8, 9, 22]. However, NCCT is easily accessible and is primarily used to exclude inappropriate acute stroke patients from undergoing futile reperfusion therapy in today’s era of endovascular thrombectomy [1, 10–12]. On the other hand, CTA is being used to select patients for endovascular treatment, as it can detect intracranial large vessel occlusion [1–3, 11]. In addition, CTA source image ASPECTS has been shown to be more sensitive than NCCT ASPECTS in the early detection of irreversible ischemic damage and more accurate in the prediction of final infarct size [15, 16]. Sallustio et al. demonstrated that CTA source image ASPECTS correlated better with follow-up ASPECTS than NCCT ASPECTS (r = 0.76 vs r = 0.51) in patients undergoing endovascular thrombectomy [15]. Kawiorski et al. showed that CTA source image ASPECTS can be a good tool to select appropriate patients for endovascular treatment; a score ≤5 predicted futile recanalization [16]. However, in those studies, it was difficult to correctly estimate overall cerebral perfusion status because it was evaluated only by early arterial phase CTA. Multiphase CTA is a more accurate evaluator of overall cerebral perfusion and collateral status [18, 23].

**Fig 2:**
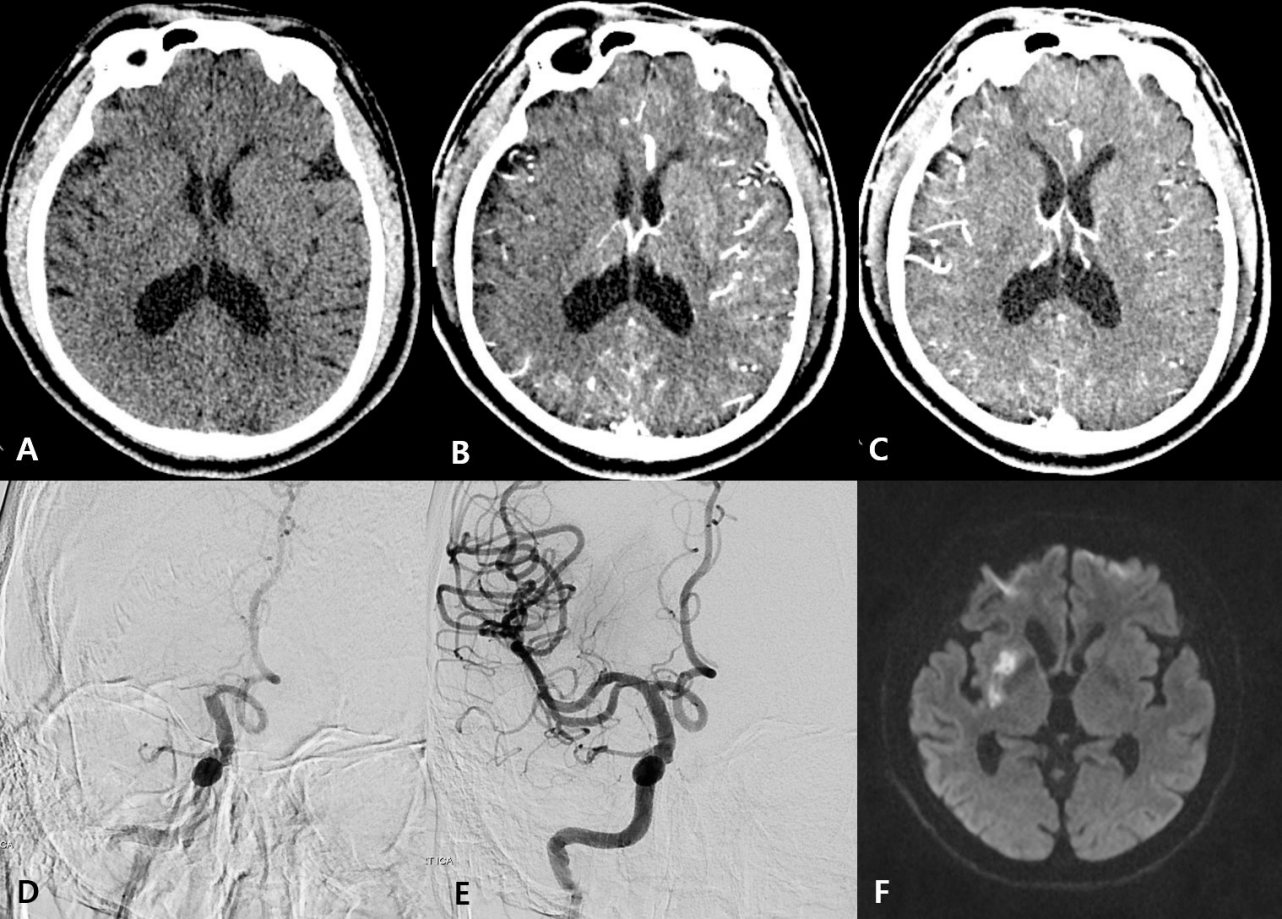
A patient in their 60s’ was admitted with acute left hemiparesis and dysarthria 45 min after symptom onset. (A) Non-contrast computed tomography showed subtle hypodensity of the right insular cortex and lentiform nucleus. (B) Arterial phase computed tomography angiography (CTA) demonstrated hypodensity involving the right insular cortex, lentiform nucleus, internal capsule, and right parietotemporal lobe. (C) Delayed phase CTA demonstrated subtle hypodensity of the right lentiform nucleus. (D) A right carotid angiogram showed an occlusion of the right middle cerebral artery. (E) The angiogram after endovascular thrombectomy showed complete recanalization of the middle cerebral artery. (F) Follow-up diffusion-weighted imaging demonstrated a small area of acute infarction in the right lentiform nucleus.

Various factors, including collateral status, are involved in maximizing endovascular treatment effectiveness and preventing futile reperfusion [24–27]. According to several randomized controlled trials, shorter time from stroke onset to reperfusion is associated with greater clinical benefit of endovascular treatment [1–6]. However, approximately 32% of patients have an unfavorable outcome even after a rapid and effective recanalization with endovascular treatment [6]. Since infarct expansion is affected by collateral status, collateral status is an important predictor of clinical outcome regardless of successful recanalization in many studies [18, 19]. Previous studies have shown that collateral status on multiphase CTA is independently associated with clinical outcome [17–19]. The delayed phase is more likely to reflect maximum collateral flow, including very delayed pial collateral flow, than the arterial phase, so CTA-DP appears to reflect final perfusion status after large vessel occlusion. Our results support this and show that CTA-DP ASPECTS highly correlates with follow-up DWI ASPECTS (Fig 2).

According to the recent DWI or CTP Assessment with Clinical Mismatch in the Triage of Wake-Up and Late Presenting Stroke Undergoing Neurointervention with Trevo (DAWN) and Endovascular Therapy Following Imaging Evaluation for Ischemic Stroke (DEFUSE 3) trials, the time window for endovascular treatment of acute stroke has been extended to 24 h [28, 29]. Patients in these studies were selected based on perfusion imaging scans using RAPID software (iSchemaView, Inc., Redwood City, CA, USA) to determine that the infarction core volume was appropriate before endovascular treatment. However, in real practice, it may be difficult for all institutions to use RAPID software. Our results suggest that CTA-DP ASPECTS is better for predicting the final infarction core than NCCT or CTA-AP ASPECTS, which may assist in patient selection for endovascular treatment.

This study has several limitations; these include its retrospective design, relatively small sample size, and lack of mortality. In addition, it only included patients with follow-up DWI; thus, patients who experienced serious neurologic deterioration that precluded follow-up MRI were excluded and selection bias may have been introduced. However, this study aimed to determine the correlation of pretreatment multiphase CTA ASPECTS with follow-up DWI, the most accurate measure of final infarction core volume. Lastly, CTA ASPECTS criteria for maximizing the therapeutic effect of endovascular therapy were not examined. Future larger multicenter studies are needed to validate the use of multiphase CTA ASPECTS in clinical decision-making.

## Conclusion

Pretreatment CTA-DP ASPECTS predicts final infarction core volume and favorable clinical outcome better than NCCT and CTA-AP ASPECTS in patients undergoing endovascular treatment of acute ischemic stroke due to anterior circulation large vessel occlusion within 6 h of symptom onset.
